# Unraveling a Three-Step Spatiotemporal Mechanism of Triggering of Receptor-Induced Nipah Virus Fusion and Cell Entry

**DOI:** 10.1371/journal.ppat.1003770

**Published:** 2013-11-21

**Authors:** Qian Liu, Jacquelyn A. Stone, Birgit Bradel-Tretheway, Jeffrey Dabundo, Javier A. Benavides Montano, Jennifer Santos-Montanez, Scott B. Biering, Anthony V. Nicola, Ronald M. Iorio, Xiaonan Lu, Hector C. Aguilar

**Affiliations:** 1 Paul G. Allen School for Global Animal Health, Washington State University, Pullman, Washington, United States of America; 2 Departamento Ciencia Animal, Universidad Nacional de Colombia, Palmira Valle, Colombia; 3 Department of Veterinary Microbiology and Pathology, Washington State University, Pullman, Washington, United States of America; 4 Department of Microbiology and Physiological Systems and Program in Immunology and Microbiology, University of Massachusetts Medical School, Worcester, Massachusetts, United States of America; 5 Food, Nutrition, and Health Program, Faculty of Land and Food Systems, University of British Columbia, Vancouver, British Columbia, Canada; Harvard Medical School, United States of America

## Abstract

Membrane fusion is essential for entry of the biomedically-important paramyxoviruses into their host cells (viral-cell fusion), and for syncytia formation (cell-cell fusion), often induced by paramyxoviral infections [*e.g.* those of the deadly Nipah virus (NiV)]. For most paramyxoviruses, membrane fusion requires two viral glycoproteins. Upon receptor binding, the attachment glycoprotein (HN/H/G) triggers the fusion glycoprotein (F) to undergo conformational changes that merge viral and/or cell membranes. However, a significant knowledge gap remains on how HN/H/G couples cell receptor binding to F-triggering. Via interdisciplinary approaches we report the first comprehensive mechanism of NiV membrane fusion triggering, involving three spatiotemporally sequential cell receptor-induced conformational steps in NiV-G: two in the head and one in the stalk. Interestingly, a headless NiV-G mutant was able to trigger NiV-F, and the two head conformational steps were required for the exposure of the stalk domain. Moreover, the headless NiV-G prematurely triggered NiV-F on virions, indicating that the NiV-G head prevents premature triggering of NiV-F on virions by concealing a F-triggering stalk domain until the correct time and place: receptor-binding. Based on these and recent paramyxovirus findings, we present a comprehensive and fundamentally conserved mechanistic model of paramyxovirus membrane fusion triggering and cell entry.

## Author Summary

The medically-important Paramyxovirus family includes the deadly Nipah virus (NiV). After paramyxoviruses attach to a receptor at a cell surface, fusion between viral and cellular membranes must occur before the virus genetic material can enter the cell and replication of the virus inside the cell can begin. For most paramyxoviruses, viral/cell membrane fusion requires the concerted actions of two viral glycoproteins. After binding to a cell surface receptor, the viral attachment glycoprotein triggers the viral fusion glycoprotein to execute viral/cell membrane fusion so the genetic material of the virus can enter the cell. However, the mechanism of this receptor-induced triggering of membrane fusion is not well understood. We identified several sequential receptor-induced structural changes in the attachment glycoprotein of NiV that are part of the viral/cell membrane fusion-triggering cascade. Importantly, we propose a mechanism of cell receptor-induced paramyxovirus entry into cells, based on the findings described here, similarities between NiV and other paramyxoviruses, and other recent advances.

## Introduction

The *Paramyxoviridae* is a medically-important negative-sense single-stranded RNA enveloped virus family that includes measles (MeV), mumps (MuV), parainfluenza (PIV), respiratory syncytial (RSV), Newcastle disease (NDV), human metapneumo- (HMPV), and the henipa-viruses Nipah (NiV) and Hendra (HeV). NiV and HeV cause high mortality rates in humans, approaching 75% in recent NiV outbreaks [1]; death is associated with syncytium formation, vasculitis, pneumonia, and encephalitis. These biosafety level 4 (BSL4) pathogens possess a broad mammalian host range [2], animal-to-human, and human-to-human transmission [1], [3], and pose bio- and agro-terrorism threats to global health and economy. Thus, NiV is classified as a category C priority pathogen in the USA NIH/NIAID research agenda.

Paramyxoviruses are generally thought to enter host cells by direct fusion of the viral and host cell membranes at physiological pH without viral endocytosis; however, recent reports for NiV and RSV suggest that they might also enter cells via macropinocytosis [4], [5]. Viral-cell membrane fusion allows release of the viral ribonucleoprotein complex into the target cell to initiate infection [6], [7]. Additionally, membrane fusion is essential for syncytium formation (cell-cell fusion), a pathological hallmark of paramyxoviral infections such as that of NiV and HeV [8] and a mechanism of cell-to-cell viral spread [3], [9], [10]. Paramyxoviral membrane fusion requires the concerted efforts of two viral proteins: the attachment (HN, H, or G) and fusion (F) glycoproteins [7]. Upon binding to its host cell surface receptor, HN/H/G triggers F to undergo a conformational cascade that merges viral and/or cell membranes. However, there is a significant knowledge gap on the mechanism(s) by which HN/H/G couples receptor binding to F-triggering [3], [9], [10].

Paramyxovirus HN/H/G and F are fairly conserved structurally. HN/H/G has a receptor-binding globular head domain comprised of a six-bladed β-barrel typical of sialidases, as shown by X-ray crystallography [11]. The HN/H/G globular head is connected to its transmembrane anchor and short cytoplasmic tail via a stalk domain. F has canonical structural/functional features of class I fusion proteins, such as an ectodomain with a hydrophobic fusion peptide and two heptad repeat regions. Upon F-triggering, F's fusion peptide is exposed and inserted into the target cell membrane to form a pre-hairpin intermediate (PHI). Subsequently, the two heptad repeats in the PHI bind each other to form a six-helix bundle (6HB), executing membrane fusion [10], [12].

Despite conservation, important differences exist among paramyxovirus attachment glycoproteins. Those of the pneumovirinae subfamily, e.g. RSV-G and HMPV-G, are significantly smaller than those of the paramyxoviridae subfamily (e.g. NiV-G or PIV5-HN). Importantly, with some exceptions (such as PIV5-HN and RSV-G only enhancing fusion, or HMPV-G not even enhancing fusion), the presence of the attachment glycoprotein is required for paramyxovirus F-triggering (reviewed in [10], [13]). HN, H, and G differ in the types of host cell receptors they recognize. While HN binds sialic acid, H and G bind protein receptors [*e.g.* MeV-H binds CD46, SLAM or nectin-4 [11], and NiV-G binds ephrinB2 or ephrinB3 [14], [15], [16]], although this is unknown for RSV-G and HMPV-G [13]. Interestingly, receptor type appears to determine how HN/H/G-F interactions modulate fusion. While *sialic acid* receptor binding appears to induce HN-F *association*, *protein* receptor binding appears to induce H-F or G-F *dissociation* of previously associated H-F or G-F complexes, suggesting that receptor type determines the mechanism of modulation of membrane fusion triggering [17], [18].

Recent studies suggest that paramyxovirus HN/H/G *stalk* domains play important roles in interacting with and/or triggering F to execute membrane fusion [11]. Recent crystal structural reports for the NDV HN ectodomain and the PIV5 HN stalk domain revealed parallel tetrameric coiled-coil stalk helical bundles (4HB) [19], [20]. Consistent with these structural data, NiV-G tetramers are important for fusion triggering, as mutation of stalk cysteine residues critical for tetramer stabilization abrogate fusion [21]. In addition, a headless (receptor-binding incapable) PIV5 HN can trigger cell-cell fusion [22], indicating that the PIV5 HN stalk is capable of triggering F. However, because PIV5 F alone can execute cell-cell fusion independent of HN [22], [23], it is uncertain whether the mechanism of PIV5 F-triggering applies to most paramyxoviruses, and particularly to those that bind protein receptors.

We found that for the protein receptor binding NiV, a headless NiV-G was sufficient to trigger NiV-F to execute cell-cell fusion without requiring receptor binding. Furthermore, molecular, biophysical, and biochemical approaches revealed that the G head prevents premature F-triggering on virions until receptor binding occurs, by concealing an F-triggering NiV-G stalk domain. Moreover, we uncovered two spatiotemporally sequential conformational steps in the NiV-G head crucial for exposure of the F-triggering stalk domain. Our results reveal a three-step receptor-induced mechanism of NiV membrane fusion triggering, and provide a comprehensive picture of the role of NiV-G in regulating such a mechanism. Combined with recent paramyxovirus findings, our data leads to a mechanistic model of F-triggering fundamentally conserved throughout the *Paramyxoviridae* family.

## Results

### A headless NiV-G can trigger NiV-F to induce cell-cell fusion without receptor-binding

**Figure 1:**
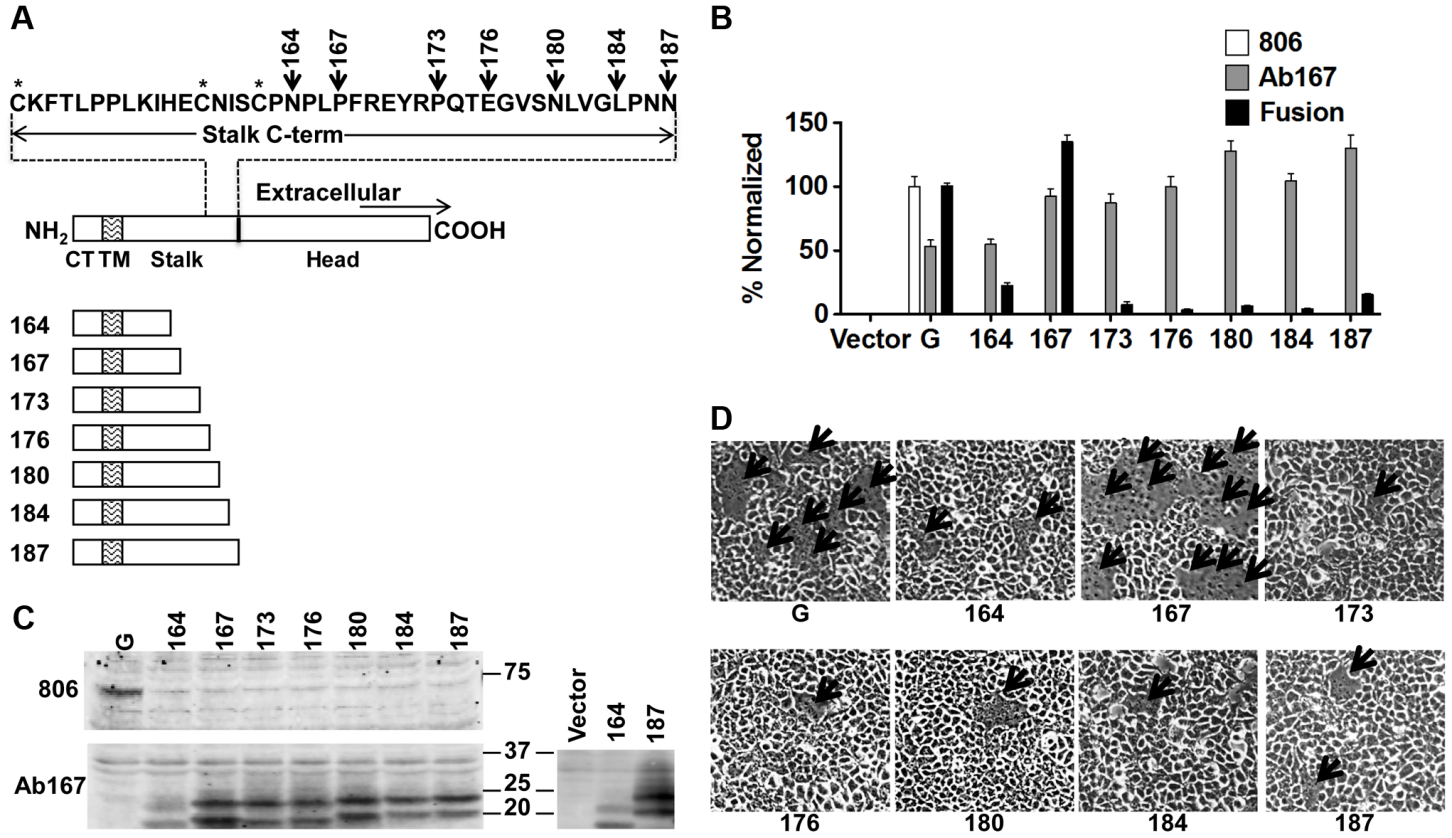
Headless NiV-G can trigger cell-cell fusion. **A**) Schematic representation of full-length wt NiV-G protein (residues 1–602), and NiV-G truncation mutants named after their most C-terminal residue. The sequence of the stalk C-terminus is shown. CT, cytoplasmic tail; TM, transmembrane domain. **B**) Relative levels of CSE measured using antibodies 806 or Ab167 by flow cytometry, normalized to wt NiV-G or mutant 167, respectively; and 293T cell-cell fusion levels induced by wt NiV-F and wt or mutant NiV-G, normalized to those of wt NiV-G. Average ± S.D. are shown. Five fields per experiment were counted, n = 3. *P* values calculated between fusion data for mutant 167 and the other mutants, or between any of the mutants and our negative control, were all <0.001, even when multiplied by the Bonferroni factor (n−1) = 7. **C**) Western blot analysis of wt NiV-G or truncation mutants blotted with 806 or Ab167. To facilitate the observation of the difference in apparent molecular weight between mutant samples, mutants 164 and 187 were run side-by side on a separate gel, shown on the right. **D**) Representative images of 293T cell-cell fusion induced by wt NiV-F and wt NiV-G or headless NiV-G mutants 17 h post transfection. Arrows point to syncytia.

Similarly to most paramyxoviruses, the presence of NiV-G and the ability of its head to bind a cell receptor (ephrinB2/B3) are required for triggering membrane fusion and viral entry [3]. To analyze the role of the NiV-G stalk in F-triggering in a biological context, we constructed seven NiV-G truncation mutants lacking the previously crystallized NiV-G head [24], [25]. The stalk-head transition is estimated to localize to residues 177–187. Our *headless* mutants 164, 167, 173, 176, 180, 184, and 187, named after their most C-terminal residue, include the entire cytoplasmic tail and transmembrane domains and either most or all of the stalk domain (Fig. 1A).

We first determined the *cell surface expression* (CSE) of these mutants. We tested recognition of the headless mutants by eight different rabbit antisera raised against full-length wild-type (wt) NiV-G or its entire ectodomain. None of these antisera reacted with the headless mutants by flow cytometry (Fig. 1B) or Western blot analysis (Fig. 1C) (example shown is antiserum 806 [26]). This suggests low antigenicity of the NiV-G stalk in the context of the full NiV-G ectodomain. However, rabbit antiserum (Ab167) raised against headless mutant 167 (Fig. 1A) reacted well with all headless mutants at the cell surface, but less efficiently with wt NiV-G by flow cytometry (Fig. 1B). Thus, it appears that the NiV-G stalk epitope(s) that Ab167 recognizes is (are) likely either directly sequestered or structurally altered by the presence of the head in full-length NiV-G.

Most importantly, with one clear exception, co-expression of wt NiV-F (F) with most mutants induced very low levels of 293T cell-cell fusion. NiV-F co-expression with mutant 167 resulted in cell-cell fusion levels *similar* to those induced by wt NiV-G/F (Fig. 1B and 1D). These data indicate that a headless NiV-G protein can trigger NiV-F *efficiently* without requiring the NiV-G head and suggest that a motif(s) in the NiV-G stalk is important and sufficient to trigger NiV-F.

**Figure 2:**
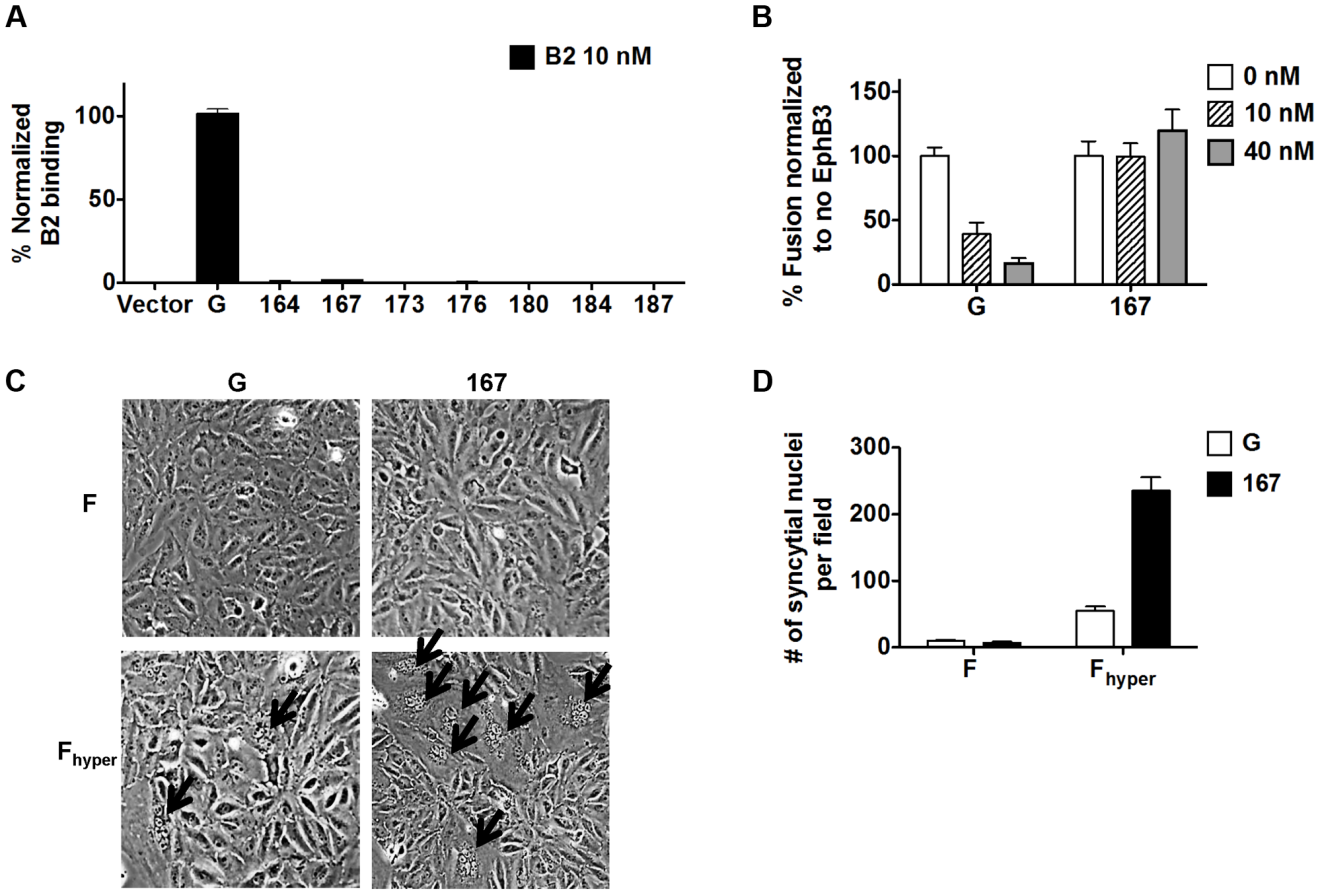
Headless fusion does not require receptor binding. **A**) Relative levels of soluble ephrinB2 receptor binding detected by flow cytometry and normalized to those of wt NiV-G. **B**) Relative levels of 293T cell-cell fusion induced by NiV-F and NiV-G or mutant 167, quantified at 18 h post transfection, in the presence of EphB3, and normalized to the level observed at 0 nM EphB3. **C**) Representative images of PK13 cell-cell fusion induced by NiV-F or NiV-F_hyper_ and NiV-G or mutant 167, 28 h post transfection. Arrows point to syncytia. **D**) Relative levels of PK13 cell-cell fusion in (C). Five fields per experiment were counted, n = 3.

Since the NiV-G *head* is known to mediate binding of NiV and HeV to their host cell receptors ephrinB2/B3 [15], [27], [28] and that receptor binding is required for fusion triggering by wt NiV-G, we tested the possibility that headless NiV-G proteins may somehow retain some ephrinB2/B3 binding ability. However, unlike wt NiV-G, none of the truncation mutants bound soluble ephrinB2 (Fig. 2A). Moreover, increasing concentrations of soluble EphB3, a natural ligand of ephrinB2/B3, inhibited wt NiV-G-induced, but not mutant 167-induced, cell-cell fusion (Fig. 2B). Lastly, when co-expressed with a previously reported hyperfusogenic NiV-F mutant (F3F5, designated F_hyper_ [29]) mutant 167 induced 4–5-fold more cell-cell fusion in PK13 cells (which express nearly undetectable levels of ephrinB2/B3 receptors [30]) than wt NiV-G (Fig. 2C–D). Altogether, these data confirm that headless NiV-G mutant 167 can trigger cell-cell fusion robustly without requiring receptor binding.

### Receptor binding to the NiV-G head exposes a stalk domain that triggers F

**Figure 3:**
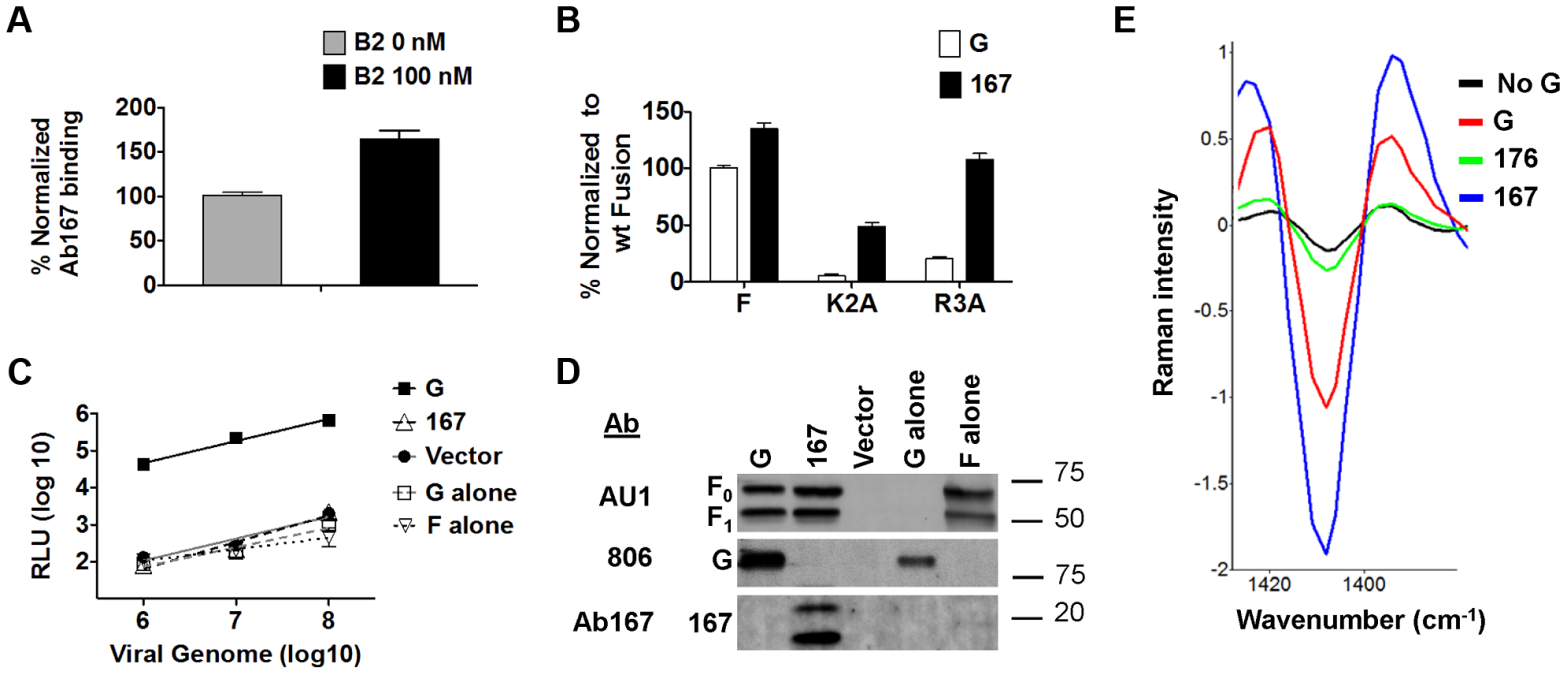
Receptor binding to the NiV-G head exposes a stalk domain that triggers F. **A**) Relative levels of Ab167 binding to PK13 cells expressing NiV-G +/− 100 nM soluble ephrinB2-Fc (B2), normalized to the level observed in the absence of B2. **B**) Relative levels of 293T cell-cell fusion induced by wt NiV-F or hypofusogenic NiV-F mutants and wt NiV-G or mutant 167, 18 h post transfection, normalized to the level induced by wt NiV-G. **C**) Relative entry levels of NiV/VSV *Renilla* luciferase reporter virions containing wt NiV-F and wt NiV-G (solid black line), wt NiV-F and mutant 167, wt NiV-G alone, wt NiV-F alone (various black or gray dashed lines), or vector alone (solid gray line). RLU were quantified 18–24 h post infection and plotted against the number of viral genomes/ml. Data shown are averages± S.D. from three independent experiments. **D**) Representative Western blot analysis of 10^9^ NiV/VSV pseudotyped virions (genome copies) from 3C showing incorporation of NiV-F and wt NiV-G or mutant 167. **E**) Second derivative transformed Raman spectral features of F glycoproteins triggered by ephrinB2-bound wt NiV-G virions (G), unbound mutant 167 virions (167), unbound control mutant 176 virions (176), or ephrinB2-bound NiV-F-alone virions (No NiV-G). The lower the 1409 cm^−1^ peak, the greater the extent of NiV-F triggering [33].

Since receptor binding is required for fusion triggering by NiV-G in the presence of the head, but not in its absence, it seems plausible that receptor binding by the head may trigger fusion by exposing a previously sequestered F-triggering stalk domain. We used anti-NiV-G stalk polyclonal antiserum Ab167 to test this hypothesis. Indeed, binding of soluble ephrinB2 to wt NiV-G enhanced the binding of Ab167 to NiV-G's stalk by about 70% (Fig. 3A). Additionally, the fact that Ab167 binds NiV-G to some extent in the absence of ephrinB2 binding suggests that either a portion of the NiV-G molecules on the cell surface are in the Ab167-binding (receptor-activated) conformation or that antiserum Ab167 binds more than one epitope in the NiV-G stalk, and that receptor ephrinB2 binding enhances exposure of only a portion of such epitopes (*e.g.* one epitope). In any event, these data indicate that ephrinB2 binding to the NiV-G head causes the exposure of at least one NiV-G stalk epitope.

We next asked whether headless mutant 167 would more readily trigger cell-cell fusion promoted by two NiV-F cytoplasmic tail hypofusogenic mutants than wt NiV-G. We previously reported that mutant K2A delays an early step, and mutant R3A a late step, in the NiV-F conformational cascade [31], [32]. We hypothesized that the loss of the head in mutant 167 would allow it to more readily trigger F, as less conformational changes would be required for mutant 167 than for wt NiV-G to trigger F, and some of these conformational changes may be energy dependent. Indeed, mutant 167 yielded 4 to 5-fold higher levels of cell-cell fusion with NiV-F hypofusogenic mutants K2A and R3A than that induced by wt NiV-G (Fig. 3B). In fact, mutant 167 rescued the levels of cell-cell fusion of mutant R3A to wt NiV-G/F levels (Fig. 3B). As our previous studies suggested that K2A and R3A need to overcome higher activation energies than wt NiV-F to undergo the conformational changes necessary to execute membrane fusion [31], [32], and that mutant R3A displays a distinct ectodomain overall conformation relative to wt NiV-F [32], the ability of mutant 167 to rescue the K2A and R3A hypofusogenic phenotypes is consistent with mutant 167 requiring less substantial conformational changes than wt NiV-G in order to trigger NiV-F. In other words, our data suggest that the loss of the NiV-G head increased the ability of the headless NiV-G to convert pre-fusion to post-fusion F, and that there likely is an energy requirement to move the head “out of the way” in order for a stalk motif to trigger F.

We next confirmed that headless mutant 167 interacts with NiV-F via a co-immunoprecipitation (co-IP) assay between wt NiV-F and wt or mutant 167 NiV-G (Fig. S1A in Text S1). These data indicated that headless NiV-G is sufficient to interact with NiV-F, but does not rule out the possibility that the NiV-G head also makes contact with NiV-F.

### Virions displaying wt NiV-F and headless NiV-G do not enter cells at least in part because F is prematurely triggered

To test whether headless NiV-G can trigger NiV-F on virions to induce viral entry (viral-cell fusion), we produced NiV/vesicular stomatitis virus (NiV/VSV) pseudotyped virions expressing a Renilla luciferase reporter gene. We previously established a quantitative, biosafety level 2 (BSL2) NiV-G/F-mediated viral entry system [26], [29], [32]. When compared at equal numbers of VSV viral genome copies over several logs of viral input, wt NiV/VSV virions entered fusion-permissive Vero cells at levels about three orders of magnitude higher than negative control bald, NiV-G-only, or NiV-F-only VSV virions. 167 virions (containing mutant 167 and wt NiV-F) did not enter cells even if spinoculation was used during infection to “force” virions into close proximity to the cell surface (Fig. 3C). Importantly, these results were not due to a lack of mutant 167 protein incorporation into NiV/VSV virions (Fig. 3D). The sharp contrast between cell-cell fusion (Fig. 1B, 1D) and viral entry (Fig. 3C) results for mutant 167 is consistent with a mechanism in which the NiV-G head prevents *premature* triggering of NiV-F by the NiV-G stalk until receptor-binding occurs.

To directly test whether NiV-F in mutant 167 virions is *prematurely* triggered, we used a confocal micro-Raman spectroscopy technique we recently developed to determine receptor-induced triggering of NiV-F embedded on the surfaces of either NiV/VSV pseudotyped virions or NiV viral-like particles expressing NiV-M/NiV-G/NiV-F [33]. In our previous study, we identified specific NiV-M, F, or G Raman spectroscopic signals, and ephrinB2 receptor binding-induced conformational changes in NiV-F detected by a change in a Raman signal at the wavenumber of 1409 cm^−1^. As shown previously, analysis of wt NiV/VSV virions pre-bound to ephrinB2 at 4°C and then incubated at 25°C for 10 min (sufficient energy and time to detect an early NiV F-triggering step) yielded a downward shift in the 1409 cm^−1^ peak, indicating a conformational change in NiV-F. Also, this NiV-F peak shift was not observed in control virions that contained only NiV-F but not NiV-G (Fig. 3E) [33]. Interestingly, mutant 167 virions unexposed to ephrinB2 exhibited higher levels of F-triggering than wt virions pre-bound to ephrinB2 (Fig. 3E), as opposed to negative control mutant 176 virions. These data indicated that independently of receptor binding, at least a subset of NiV-F molecules in mutant 167 virions is prematurely triggered.

### Monoclonal anti-NiV-G antibodies neutralize two distinct conformational epitopes in the NiV-G head

**Figure 4:**
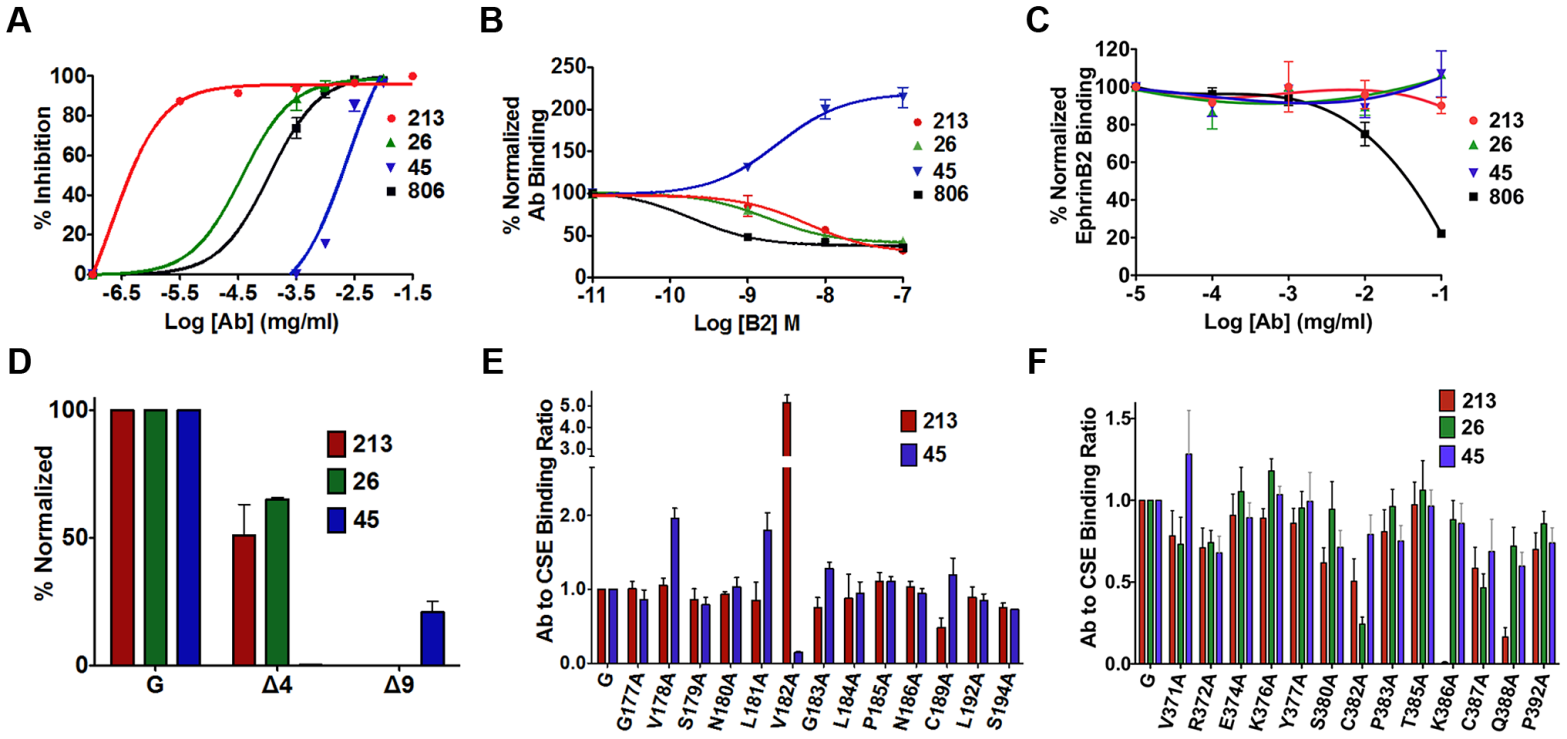
Mapping of two distinct neutralizing Mab epitopes in the NiV-G head. **A**) Neutralization curves of polyclonal (806) and monoclonal antibodies (Mab) (26, 45, 213) against wt NiV/VSV pseudotyped virions. Averages +/−S.D. are shown, n = 3. **B**) Antibody binding to CHO cells expressing NiV-G, in the presence of increasing soluble ephrinB2 concentrations, measured by flow cytometry, and normalized to mean fluorescence intensity (MFI) values at 0M ephrinB2. Average ± S.D. are shown, n = 3. **C**) EphrinB2 binding in the presence of increasing antibody concentrations, and normalized to MFI values in the absence of antibody. n = 4. **D**) Mab binding to region 4 (Δ4) and 9 (Δ9) deletion mutants, measured by flow cytometry as in (B). MFI values were normalized to wt NiV-G values for each respective Mab. Average ± S.D. are shown, n = 3. **E**) Mab213 and Mab45 binding to the region 4N mutants as in (B), but normalized to each protein's CSE levels (Mab anti-HA). Average ± S.D. are shown, n = 3. Mutants that did not express at the cell surface were excluded. **F**) Mab213, Mab26, and Mab45 binding to region 9 mutants. Experimental procedures were similar to those in (E), n = 8.

Based on the idea that receptor binding induces the exposure of an F-triggering domain in the NiV-G stalk, we explored the changes in the head that must take place to link these two events. We previously identified a receptor-induced conformational change in NiV-G, marked by enhanced binding of anti-NiV-G monoclonal antibody 45 (Mab45) and by secondary structural changes in NiV-G [34]. Here, we analyzed the ability of several neutralizing anti-NiV-G Mabs (Fig. 4A) to detect conformational changes in NiV-G. We observed receptor binding *enhanced* Mab45 binding to NiV-G (as expected), but *decreased* binding of Mab213 and Mab26 to NiV-G, in a receptor concentration-dependent manner (Fig. 4B). These data suggest that either Mab213 and Mab26 both detect a receptor induced conformational change in NiV-G, or they compete for binding to NiV-G with soluble ephrinB2. We tested the latter scenario and observed that neither Mab213 nor Mab26 (up to [0.1 mg/ml]) blocked binding of NiV-G to ephrinB2 (Fig. 4C). Altogether, these data indicate that ephrinB2 binding to NiV-G induces a conformational change in NiV-G that Mab213 and Mab26 can detect [ostensibly the hiding of an epitope(s)].

We then attempted to map the binding epitopes of Mab45, Mab213 and Mab26 in NiV-G. We first used a series of well-expressed NiV-G deletion mutants [15], [34]. While deletion of region 4 (residues 195–211, mutant Δ4) in the NiV-G head abolished binding of Mab45 to NiV-G, deletion of region 9 (residues 371–392, mutant Δ9) abolished binding of Mab213 and Mab26 to NiV-G (Fig. 4D). The locations of regions 4 and 9 in NiV-G relative to the ephrinB2 cell-receptor binding site are shown (Fig. S3). We previously showed via triple alanine (Ala) scan mutagenesis that Mab45 does not directly bind region 4 [34]. Thus, to map the binding epitope of Mab45 in NiV-G, we mutated and analyzed a region C-terminal from the stalk domain and N-terminal from region 4 (region 4N, residues 177–194), by Ala scan mutagenesis using a C-terminal HA-tagged version of NiV-G. We similarly analyzed region 9 to map the binding epitope(s) of Mab213 and Mab26 (Fig. S2 in Text S1 & 4E–F). We previously showed that HA-tagged NiV-G functions and binds ephrinB2 well [15], [34].

We then compared by flow cytometry the binding levels of Mab45 and Mab213 to each Ala scan mutant in region 4N normalized to each mutant's CSE levels, as measured by anti-HA-tag antibody binding (Fig. 4E). Although mutant V182A bound Mab213 at higher than wt levels, it bound Mab45 hardly at all, indicating that residue V182 in NiV-G is important for Mab45 binding. Interestingly, three mutants in region 4N: V178A, L181A, G183A, and C189A displayed enhanced binding levels to Mab45, a phenotype we previously showed corresponds to a NiV-G “post-receptor-binding” conformation [21]. Similar analysis of region 9 revealed that residues K386 and Q388 in NiV-G are important for NiV-G binding to Mab213, while residue C382 is important for its binding to Mab26 (Fig. 4F). Thus, regions 4N and 9 contain binding residues for antibodies Mab45 and Mab213/Mab26, respectively, and Mab45 binds a different NiV-G epitope than Mab213/Mab26, making it likely that Mab213 and Mab26 detect a distinct conformational change from that detected by Mab45.

### Two NiV-G head epitopes modulate receptor-induced membrane fusion

**Figure 5:**
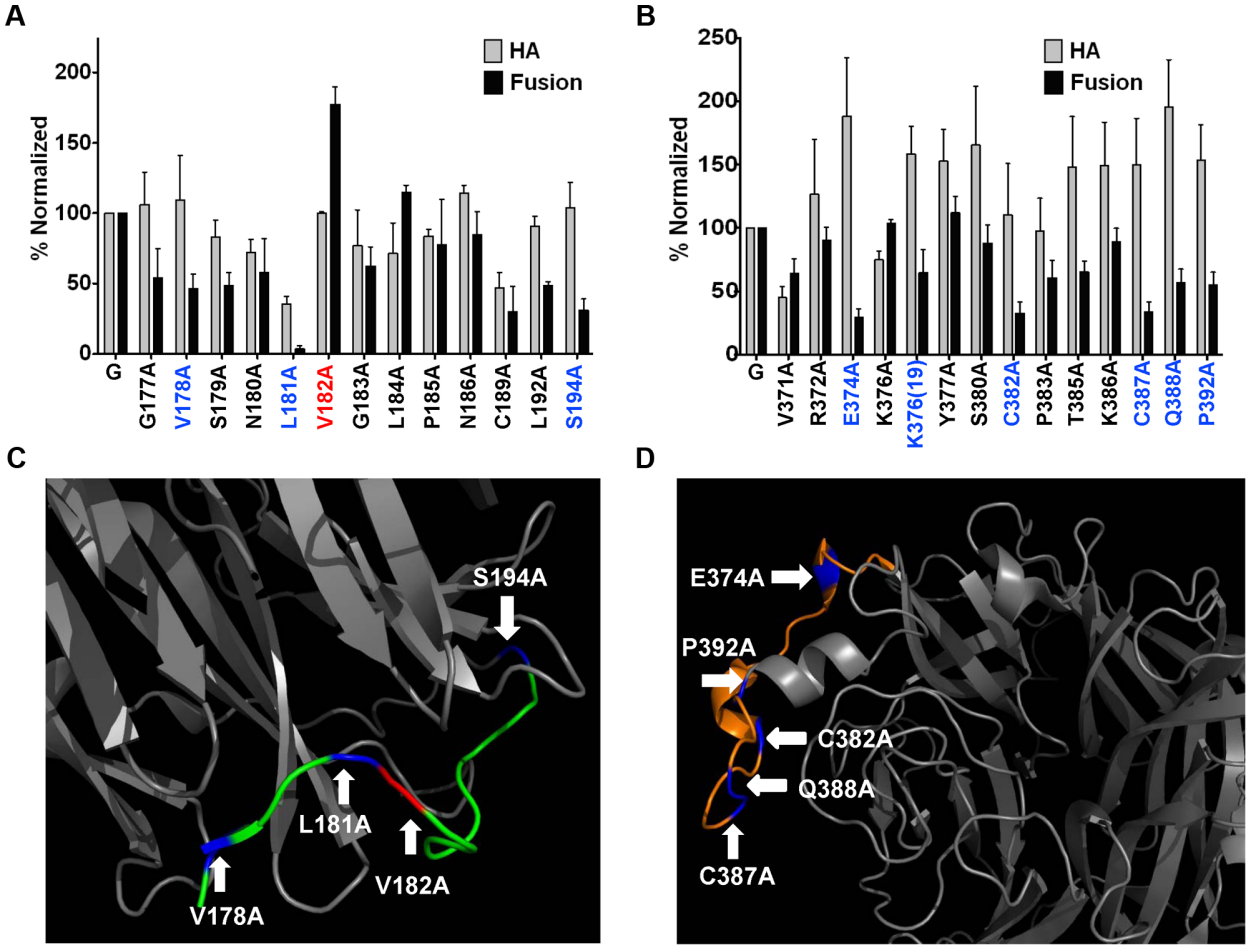
Both regions 9 and 4N modulate NiV membrane fusion. CSE (HA) and cell-cell fusion levels of region 4N (**A**) and 9 (**B**) mutants. CSE levels were measured in 293T cells as in Fig. 4E. 293T cell-cell fusion levels induced by wt NiV-F and wt or mutant NiV-G, normalized to values of wt NiV-F/G. n = 3–8. **C) & D)** Depictions of regions 4N (C), or 9 (D), from the crystalized NiV-G head structure in Fig. 4G. Blue and red colored residues mark hypo- or hyper-fusogenic mutants, respectively.

As Mab45, Mab213, and Mab26 neutralize NiV-G (Fig. 4A) without blocking receptor binding (Fig. 4C), we tested whether these Mabs bind NiV-G epitopes in regions 4N and 9 important for membrane fusion modulation. Normalization of each point mutant's levels of cell-cell fusion and CSE to wt NiV-G levels, and calculation of fusion indexes as normalized fusion/normalized CSE (fusion index for wt NiV-G = 1, Fig. S2 in Text S1), revealed that region 4N mutants V178A, L181A, and S194A yielded *hypo*fusogenic phenotypes (fusion indexes <0.5), while mutant V182A and to a lesser extent mutant L184A yielded *hyper*fusogenic phenotypes (fusion indexes >1.5, Fig. 5A & S2 in Text S1). Additionally, region 9 mutants E374A, C382A, C387A, Q388A, and P392A yielded hypofusogenic phenotypes (Fig. 5B & S2 in Text S1). Mutations in regions 4N and 9 that yielded *hyper-* (red) or *hypo-* (blue) *-fusogenic* phenotypes are shown in the NiV-G head structure, respectively (Fig. 5C–D). These results indicate that, as hypothesized, regions 4N and 9 modulate membrane fusion.

### Sequential three-step mechanism of NiV membrane fusion triggering

**Figure 6:**
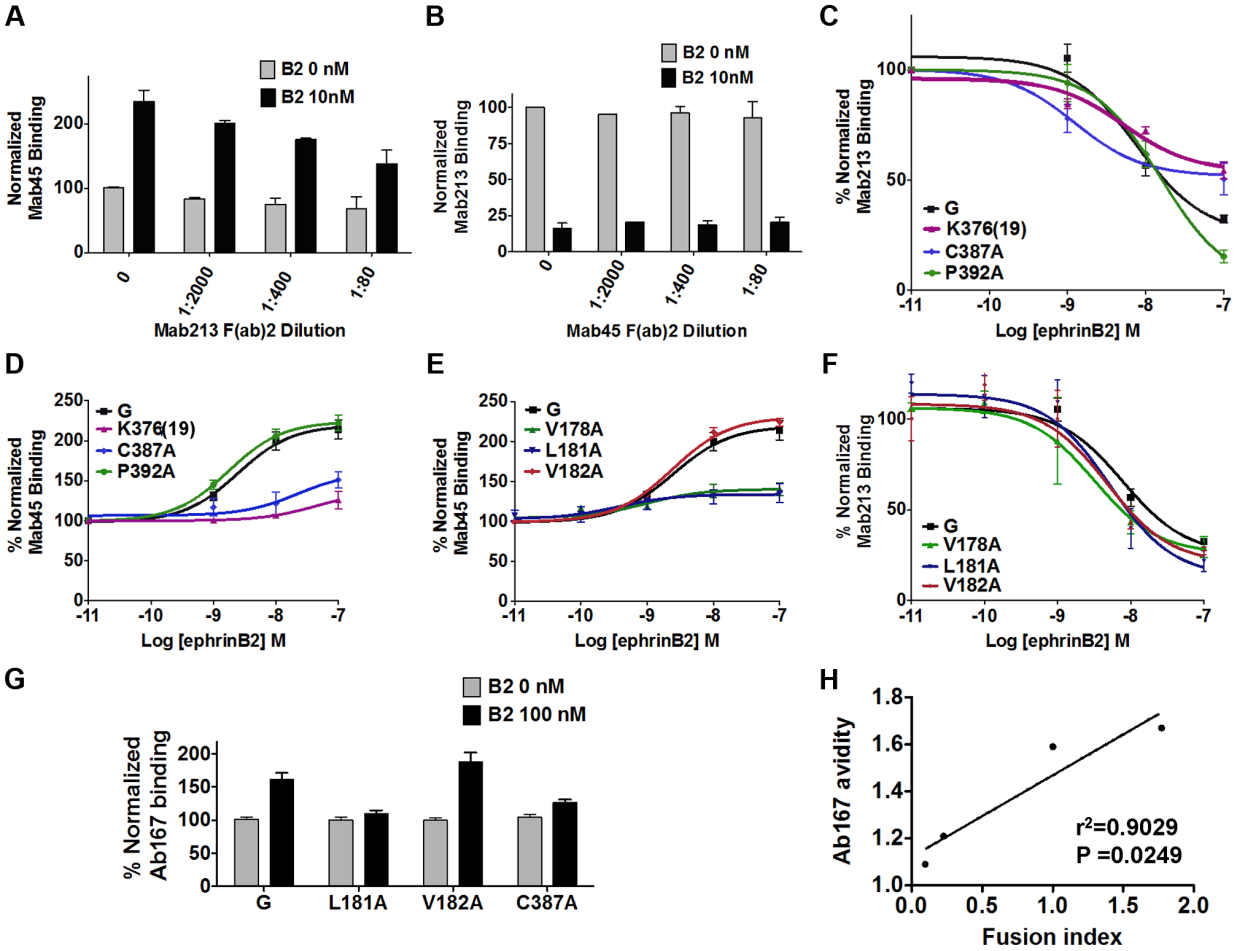
Spatiotemporal sequence of three conformational steps that trigger F. **A**) Binding of Mab45 in presence of increasing concentrations of Mab213 F(ab)2. **B**) Binding of Mab213 in presence of increasing concentrations of Mab45 F(ab)2. **C**) Mab213 binding to region 9 mutants in the presence of 0, 1, 10, and 100 nM ephrinB2, measured by flow cytometry and normalized to MFI values at 0 nM ephrinB2. Average ± S.D. are shown. Similarly, **D**) Mab45 binding curve for region 9 mutants, **E**) Mab45 binding curves for region 4N mutants, and **F**) Mab213 binding curves for region 4N mutants. **G**) Relative levels of Ab167 binding to PK13 cells expressing NiV-G or selected fusion mutants, pre-bound to 0 nM or 100 nM soluble ephrinB2 (B2) at 4°C, normalized to values obtained at 0 nM B2. **H**) The ratio of Ab167 binding avidities at 100 nM/0 nM B2 to NiV-G in (G) were plotted against the fusion/CSE rations (fusion index) for each respective mutant, obtained from Fig. S2 in Text S1, using GraphPad PRISM.

We then analyzed the spatiotemporal relationship between the two receptor-induced conformational changes detected in the NiV-G head by Mab45 and Mab213/Mab26, using Mab45 and Mab213 F(ab)2 fragments undetectable by the secondary antibodies that detect their full-length counterparts. We tested whether pre-binding region 9 with Mab213 F(ab)2 would inhibit the receptor-induced conformational change in region 4N detected by Mab45, and vice-versa. While Mab213 F(ab)2 reduced the receptor-induced conformational change detected in region 4N (enhanced binding of Mab45 decreased, Fig. 6A), Mab45 F(ab)2 did not inhibit the conformational change detected in region 9 by Mab213 (decrease in Mab213 binding remained unchanged, Fig. 6B). These data are consistent with: 1) Mab45 and Mab213/Mab26 binding distinct epitopes in NiV-G, 2) the conformational change in region 9 occurring spatiotemporally prior to that in region 4N (our theorem), and 3) region 9 being relatively closer to the receptor-binding site in NiV-G (Fig. S3 in Text S1).

To further test our theorem, we analyzed highly hyper- or hypo-fusogenic mutants in regions 4N and 9 for their abilities to undergo the conformational changes in regions 9 and 4N detected by Mab213 and Mab45, respectively. Additionally, to fully disrupt the presumptive upstream conformational change in region 9, we constructed and tested a region 9 mutant with an insertion of a random 19-residue peptide at K376 [K376(19)] (Fig. S2 in Text S1). As opposed to region 9 mutant P392A, hypofusogenic mutants C387A and K376A(19) had reduced conformational changes in region 9 (Fig. 6C). Consistent with our theorem, both C387A and K376(19), but not P392A, also had reduced enhancement of receptor-induced Mab45 binding in region 4N (Fig. 6D). Additionally, two region 4N hypofusogenic mutants (V178A and L181A) displayed little to no conformational changes in region 4N, as opposed to wt NiV-G or hyperfusogenic mutant V182A (Fig. 6E). However, consistent with our theorem, no region 4N mutants exhibited a decrease in the conformational change detected in region 9, as detected by Mab213 binding (Fig. 6F). In summary, our data are consistent with the receptor-induced conformational change in region 9 occurring prior to that in region 4N.

Next we tested whether regions 4N and 9 affect the downstream exposure of the NiV-G stalk domain by testing the ability of the most hyper- or hypo-fusogenic mutants in regions 4N and 9 to undergo receptor-induced exposure of the Ab167 G stalk domain. Fusogenicity of the region 4N and 9 mutants directly correlated with their ability to undergo receptor-induced exposure of the NiV-G stalk domain (Fig. 6G–H), consistent with NiV-G stalk domain exposure-induced F-triggering occurring relatively downstream of changes in the NiV-G head.

**Figure 7:**
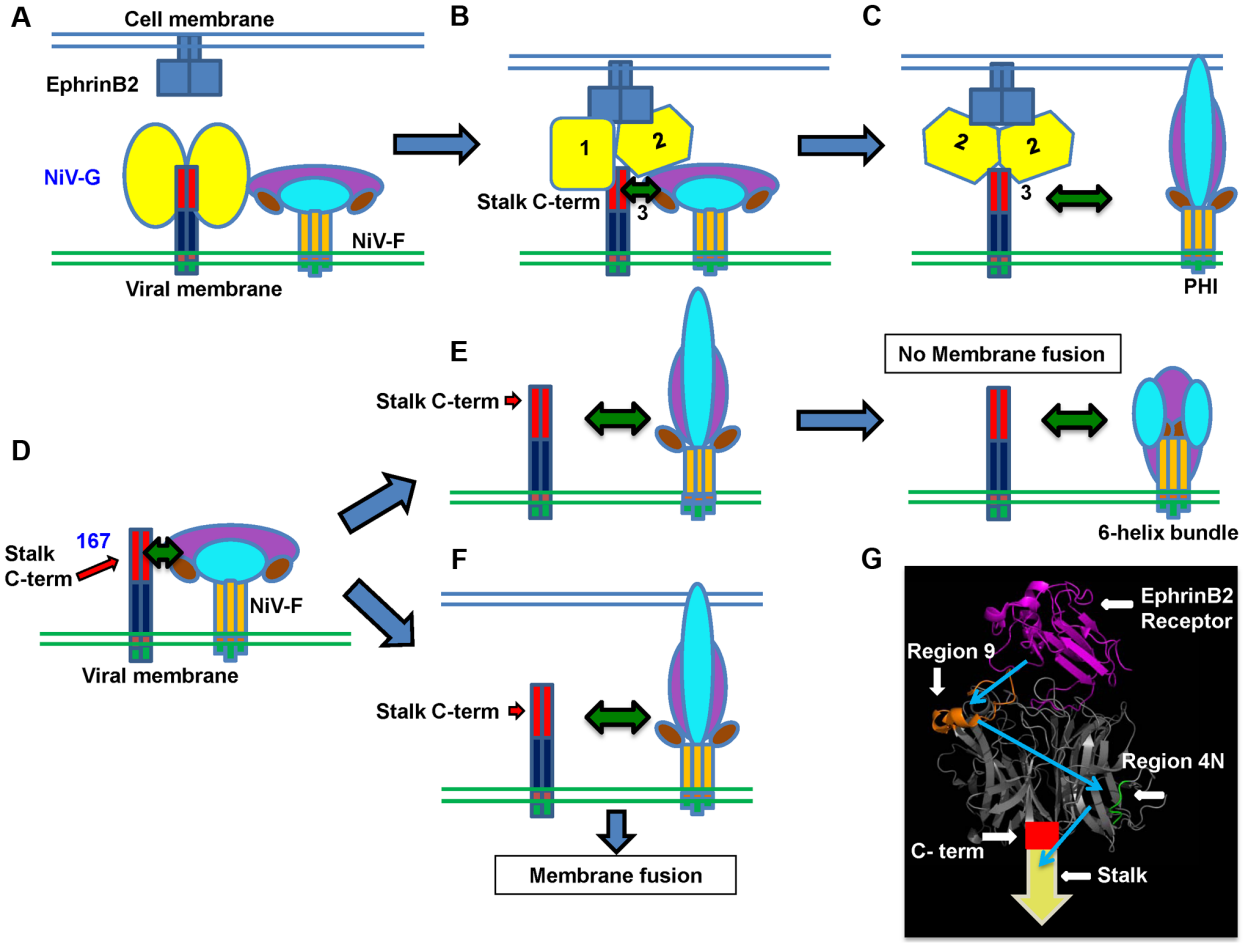
Mechanistic model of henipavirus membrane fusion triggering. **A**) The NiV-G stalk C-terminal region (C-term) that triggers F is covered by the NiV-G head(s) previous to ephrinB2 receptor binding. **B**) Upon receptor binding, the G head(s) undergoes at least two conformational changes that result in exposure of the G stalk C-term, which triggers F to undergo conformational changes that result in **C**) dissociation of F from G and membrane fusion. **D**) In the case of mutant 167, either: **E**) In the case of virions, NiV-F will be prematurely triggered in the absence of a target membrane, resulting in no viral entry; or: **F**) In the case of cell-cell fusion, in the presence of a target membrane, NiV-F will execute membrane fusion. The NiV-G head (yellow), stalk C-term (red), stalk N-term (dark blue), transmembrane domain (orange), cytoplasmic tail (green), NiV-F trimer head (purple, cyan, and brown), stalk (dark yellow), ephrinB2 receptor (blue), are shown. **G**) PyMOL representation of NiV-G head bound to its ephrinB2 receptor (magenta). Region 9 (orange) and 4N (green) are shown. The crystallized structure was taken from [24] (PDB 2VSM). The spatiotemporal receptor-induced sequence of events in NiV-G are shown in blue arrows.

Last, we tested whether any of the phenotypes observed were due to altered abilities to bind the cell receptor ephrinB2 or NiV-F. Relative to their levels of CSE, only mutant C387A had a slight, and mutant K376(19) a considerable, reduction in receptor ephrinB2 binding abilities (Fig. S3 in Text S1). In addition, all mutants analyzed bound NiV-F at roughly wt NiV-G levels, except for L181A, which bound NiV-F at greater than wt NiV-G levels (Fig. S1B in Text S1). Overall, our results indicate that: decreasing receptor binding [mutant K376(19)] decreased both region 9 and region 4N conformational changes; blocking region 9 blocked the region 4N conformational change; and blocking region 4N did not block the region 9 conformational change. Combined, these results indicate the following spatiotemporal order of three events: receptor binding, followed by 1) region 9 conformational change, 2) region 4N conformational change, and 3) G stalk domain exposure and F-triggering (Fig. 7G).

### A model for the mechanism of receptor binding-induced NiV membrane fusion

Based on all these findings, we propose the spatiotemporal F-triggering model depicted in Figure 7. In the presence of the G head, the G stalk C-terminal F-triggering domain (red) is relatively “hidden” by other NiV-G sequences in the NiV-G head N-terminus and/or stalk C-terminus (Fig. 7A). However, cell receptor binding to the NiV-G head causes conformational changes in the head (Fig. 7B) that allow exposure of a G stalk C-terminal domain (Fig. 7C) that can now interact with and trigger F to execute membrane fusion. In contrast, in virions expressing headless mutant 167, the NiV-G stalk C-terminal F-triggering domain is always exposed (Fig. 7D), triggering NiV-F prematurely, as virions exist in supernatants at 37°C for long periods of time prior to their collection, thus eliminating viral infectivity (Fig. 7E). During cell-cell fusion, nearby target membranes are available when F is triggered, and thus 167 induces cell-cell fusion (Fig. 7F).

## Discussion

Our results reveal a comprehensive mechanism of cell receptor-induced NiV membrane fusion triggering. Upon ephrinB2 binding, the NiV-G head undergoes at least two conformational steps in the head that result in exposure of a NiV-G stalk domain that interacts with and triggers F (Fig. 7). To our knowledge, this is the most detailed mechanistic molecular picture of paramyxovirus attachment protein linking receptor binding to F-triggering to date. Additionally, the headless NiV-G prematurely triggers F in virions, causing the loss of viral entry (Fig. 3), implying a crucial role for the NiV-G head in preventing premature F-triggering by a G stalk domain until receptor binding occurs.

NiV-G/F interactions at viral or cell surfaces prior to receptor binding are well documented [3]. In addition, the first step of F-triggering, the transition time between F's pre-fusion and pre-hairpin intermediate conformations, occurs rather rapidly, with a half-life of ∼4 min [31]. Thus, pre-formed NiV-G/F complexes likely increase the efficiency of G-stalk/F interactions upon receptor binding and prior to G/F dissociation. Evidently, G/F interactions alone are insufficient for F-triggering and must somehow change for F-triggering to occur. It has previously been reported that: 1) a NiV-G mutant that lacks the C-terminal stalk domain 146–182 (Δ3), but retains the head and stalk N-terminal region, co-IP's with NiV-F [21] and 2) NiV-G mutants with added N-glycans to N-terminal stalk region 75–133 do not prevent G/F interactions [35]. Based on these findings and our results reported here, we speculate that bi-dentate G-F interactions involving both the NiV-G head and possibly the stalk N-terminus occur prior to receptor binding, and that either of these regions is sufficient to provide a detectable interaction with NiV-F. Full understanding of the residues involved in changes in these G/F interactions during membrane fusion will likely identify new potential targets for therapeutic intervention for the paramyxoviruses including NiV.

It was recently reported that a headless PIV5 HN (strain W3A) can trigger PIV5-F, suggesting that a HN stalk hidden by the HN heads is exposed upon sialic acid receptor binding [22]. Notably, PIV5 W3A F alone can induce cell-cell fusion at 37°C and 42°C, but the presence of HN enhances fusion [22], [23]. In contrast, for NiV and for most paramyxoviruses, wt F or even hyperfusogenic F mutants are unable to trigger membrane fusion in the absence of HN/H/G [29], [32]. Another major functional difference between PIV5 and NiV is the type of receptors they bind, as sialic acid *vs.* protein receptor binding appears to modulate fusion via PIV5 HN-F *association* or NiV G-F *dissociation*, respectively [3], [36]. Here, we report that a paramyxovirus attachment protein strictly required for membrane fusion can trigger F in the absence of its receptor binding head. Additionally, it was recently reported that a stabilized headless measles virus H stalk can trigger F [37]. Thus, our NiV results (Figs. 1–3) combined with those for PIV5 [22] and MeV [37] suggest that despite the differences between PIV5 and NiV just described, fundamental aspects of the mechanism of membrane fusion triggering are conserved throughout the paramyxovirus family. However, as described in our introduction, within the paramyxoviridae family there are significant differences between the attachment glycoproteins of the paramyxovirinae and pneumovirinae subfamilies, thus it is possible that our three-step model of receptor-induced fusion triggering applies to the *Paramyxovirinae* but not to the *Pneumovirinae* subfamilies.

Despite this apparent conservation, specifics on the receptor-induced mechanism of activation of HN/H/G may vary among paramyxoviruses [13]. Two recent reports indicate that rearrangement of the MeV H tetramer is crucial for F-triggering [38], [39]. Oligomeric rearrangements of rigid monomers may possibly account for our results for NiV-G presented here; however, it is also possible that the mechanism of receptor-induced activation of NiV-G involves some level of secondary structural changes. Although no structural differences were observed between a soluble NiV-G head monomer bound or unbound to the ephrinB2 receptor, it is important to recognize that this study utilized only the NiV-G head, lacking the NiV-G stalk [25]. We previously reported, using circular dichroism (CD), that receptor-induced secondary structural changes take place in a soluble NiV-G full ectodomain protein, but not in a soluble NiV-G head (lacking the stalk domain) [34]. Thus the stalk may be necessary to maintain NiV-G in the pre-receptor-activated conformation, and the lack of the stalk may result in the soluble NiV-G head converting prematurely to the receptor-activated conformation. Alternatively, the receptor-induced secondary structural changes in the NiV-G ectodomain detected by CD may occur solely in the stalk. In this case, the changes in Mab213 and Mab45 recognition we observed in the head may be purely a result of oligomeric rearrangements of rigid monomers. Mab213 and Mab45 bind two distinct NiV-G head epitopes located on different faces of the NiV-G monomer (Fig. 7G) making it less likely that oligomeric rearrangements of rigid head monomers solely account for our results. A combination of a future structure of the NiV-G tetramer and our knowledge of the binding epitopes of Mab213 and Mab45 provided here should help us distinguish between these possibilities.

Although previous NiV studies uncovered G stalk mutations that modulate fusion, the domain(s) in NiV-G that trigger F have remained unknown. Our findings provide conclusive evidence that the NiV-G stalk is sufficient to trigger NiV-F. The precise residues in the NiV-G stalk exposed upon receptor binding and capable of F-triggering are yet to be determined. The finding that 167, but neither 164 nor 173, efficiently triggered F, though all three constructs were efficiently expressed (Fig. 1B–D), suggests that the G stalk C-terminal residues 164–167 are important for F-triggering. Consistent with this, a recent NDV ectodomain crystal structure suggested that an equivalent stalk C-terminal region likely interacts with the HN head [20]. Thus, it was recently proposed that HN transitions from *heads down* to *heads up* conformations, exposing the stalk C-terminal F-triggering domain [22]. Our results are consistent with that model and further indicate that two conformational changes in the NiV-G head are necessary for the exposure of the stalk F-triggering domain. Furthermore, Ab167, raised against mutant 167, binds at least one epitope exposed after receptor binding, consistent with residues N-terminal of 167 being important for F-triggering.

None of the antisera made against the full-length NiV-G ectodomain recognized the headless mutants tested, neither at the cell surface nor by Western blot analysis (Fig. 1). However, Ab167, produced against mutant 167, recognized the headless mutants, suggesting that the G stalk is somewhat immunogenic, and that the immunogenic stalk region(s) is relatively hidden in full-length NiV-G. The question arises: why are NiV-G mutants longer than 167 incapable of triggering NiV-F efficiently? They contain the domain that ostensibly triggers F, present in mutant 167, and they are expressed efficiently (Fig. 1B–D). We speculate that residues C-terminal of 167 render the headless stalk more stable and thus unable to undergo a conformational change, possibly involving the unfolding of the 4HB stalk, to make it F-interactive. In other words, it is possible that the stalk needs to unravel in order to bind to and trigger F and that segments that extend beyond 167 are unable to undergo such unraveling until signaled by head residues upon receptor binding. Another possibility is that residues C-terminal from 167 fold over and directly “cover” residues N-terminal of 167. However, the fact that the longer headless constructs are recognized well by Ab167 argues against this possibility.

Overall, our data imply that wt NiV-G undergoes a receptor-induced conformational cascade that includes two sequential conformational steps in the head that result in exposure of a C-terminal stalk domain that triggers F to undergo its own conformational cascade. In contrast, the headless mutant 167 does not need to undergo such changes, triggering NiV-F either highly efficiently or prematurely, depending on whether F is at the cell or viral surface. These findings lead us to a mechanistic model of cell receptor-induced NiV and paramyxovirus membrane fusion and viral entry triggering (Fig. 7).

## Materials and Methods

### Expression plasmids

Expression plasmids for codon-optimized NiV-G and NiV-F genes, tagged or untagged at their C-terminus with the HA or AU1 tags, respectively, were previously described [40], as was hyperfusogenic NiV-F mutant F3F5 [29]. The NiV-G headless mutants were constructed by inserting two stop codons (TAA TAA) at the corresponding sites.

### Cell culture

CHO (CHOpgsA745) and Vero cells were cultured in Minimal Essential Medium alpha with 10% fetal bovine serum (FBS). PK13 and 293T cells were cultured in Dulbecco's Modified Eagle's medium with 10% FBS.

### Rabbit antiserum production

Rabbit antiserum (Ab167) was produced by co-expressing NiV-M and headless NiV-G mutant 167 (NiV-G sequences 1–167) using corresponding expression plasmids at a 1∶1 ratio in rabbits (three DNA boosts). NiV-M was included to produce viral-like particles (VLPs) that incorporate mutant 167 protein *in vivo* [41]. Anti-NiV-G and anti-NiV-F polyclonal antisera or Mabs were produced previously in a similar fashion [15], [29], [32], [40].

### Detection of cell surface expression, ephrinB2, and antibody binding using flow cytometry

Binding of anti-NiV-G specific rabbit antibodies, mouse anti-HA Mab, or soluble ephrinB2 (B2-hFc, R & D Systems), to cell surface NiV-G, were measured by flow cytometry. Primary antibodies were used at 1∶100 to 1∶1,000 dilutions, and B2-hFc at 10 nM, and PE-conjugated secondary antibodies (Caltag) at 1∶1000 dilution. Background mean fluorescence intensity (MFI) obtained by binding equal concentrations of primary and secondary reagents to PCDNA3.1 (mock)-transfected 293T cells was subtracted from the MFI of NiV-G expressing 293T or PK13 cells as indicated.

### Quantification of syncytia

NiV-F and wt or mutant NiV-G expression plasmids (1∶1 ratio, 2 µg total) were transfected into 293T or PK13 cells (∼80% confluency), grown in 6-well plates. Cells were fixed in 0.5% paraformaldehyde 16–24 h post-transfection. To account for both syncytium size and numbers, cell-cell fusion was quantified by counting the number of nuclei within syncytia per 200X field (5 fields were counted per well per experiment). Syncytia were defined as 4 or more nuclei visualized within a common cell membrane [29], [40].

### Western blot analysis

Cells or pseudotyped NiV/VSV-rLuc virions expressing NiV-G and or -F were lysed in RIPA buffer (Cell Signaling Technology) and protease inhibitors (cOmplete, Mini, Roche). Cell or viral lysates were then subjected to SDS-PAGE and NiV-G or NiV-F glycoproteins were subsequently detected by Western blotting using rabbit Ab167 or 806 or mouse anti-HA or anti-AU1 antibodies (Caltag), at 1∶100–1∶3000 dilutions. Fluorescent secondary antibodies (Li-Cor Biosciences) were used at a dilution of 1∶10,000, respectively, and detected with a Li-Cor Odyssey fluorimager.

### Quantitation of viral entry, viral genome copies, and viral neutralization

NiV/VSV pseudotyped virions expressing the *renilla* Luc reporter gene were produced as previously described [15], [16], [29]. Virions from viral supernatants collected 28 h post-infection were purified over a 20% sucrose cushion. Vero cells plated in 96-well were infected with the NiV/VSV-rLuc virions in infection buffer (PBS+1% FBS) for 2 h at 37°C over several logs of viral dilution. After 2 hours, Vero cell growth medium was added. 18–24 h post-infection, cells were lysed, and luciferase activity was measured in relative light units (RLU) using a renilla luciferase detection system (Pierce) and an Infinite M1000 microplate reader (Tecan Ltd). Quantitation of viral genome copies was previously described [29]. RLU were plotted against genome copies per milliliter and regressed using GraphPad Prism.

### Co-immunoprecipitation

Approximately 20 hours post-transfection, 293T cells were washed with PBS and lysed in RIPA buffer (Millipore) supplemented with complete protease inhibitor (cOmplete Mini, Roche). Cellular debris was pre-cleared by centrifugation and whole cell lysates (supernatants) were incubated with either 50 ul of µMACS anti-HA MicroBeads (Miltenyi Biotec) or 50 µl of polyclonal rabbit Ab167 and 100 ul of protein G MicroBeads) for 30 min. at 4°C with rotation and then purified and eluted over μ columns (Miltenyi Biotec) following the manufacturers protocol. RIPA buffer was used for all wash steps. Cell lysate (10% of a well from a 6-well plate) and purified elutions (45%) were separated by either 10 or 12% PAGE to separate full-length G mutants or deletion mutants respectively. F and G proteins were detected by Western blot analysis as indicated above, using the indicated antibodies.

### Raman spectroscopy

Procedures were identical to those fully described in [33].

## Supporting Information

Text S1 **S1**) Co-immunoprecipitation between NiV-F and selected headless NiV-G and point mutants from regions 4N and 9. 293T cells were transfected with indicated plasmids. Cell lysates were subjected to immunoprecipitation to pull down (A) wt NiV-G (G) or headless NiV-G mutant 167 using polyclonal rabbit Ab167 antibody (IP: Ab167) or (B) wt NiV-G (G) or selected NiV-G mutants in regions 4N and 9 (V178A, L181A, V182A, C387A), using HA-linked magnetic MicroBeads (IP: αHA). Immunoprecipitated G or G mutants were then detected by immunoblotting using either Ab167 (IB: Ab167) (A) or rabbit anti-HA (IB: αHA) (B) antibodies. Uncleaved(F_0_) and cleaved version of F (F_1_) were detected using mouse anti-AU1 (IB: aAU1). Proteins were detected from total (left panels) and from immunoprecipitated (right panels) cell lysates. In agreement with published reports, we observed a doublet of NiV-G when using antiserum 806 or anti-tag antibodies, when the SDS-PAGE bands were sufficiently resolved, likely resulting from variable NiV-G secondary modifications such as glycosylation. **S2**) Summary of the alanine scan mutants for regions 4N and region 9. Mutants were generated using a QuikChange ™ site directed mutagenesis kit (Stratagene). Fusion indexes = ratio of % cell-cell fusion levels normalized to NiV-G/% CSE levels normalized to those of wt NiV-G. N.D. = non determined because CSE was too low to be reliable. **S3**) Pymol representation of NiV-G head bound to its ephrinB2 receptor (magenta). Region 9 (orange) and 4N (green) are shown. The crystallized structure was taken from [24] (PDB 2VSM). **S4**) Cell surface expression and ephrinB2 binding of fusion mutants in regions 4N and 9. Mutants expressing notably hypofusogenic or hyperfusogenic phenotypes were selected and analyzed. CHO cells were transfected with either wt or mutant NiV-G expression plasmids. Binding of soluble ephrinB2 to transfected cells expressing NiV-G, along with cell surface expression using an anti-HA Mab, were measured by flow cytometry. Average ± S.E. are shown. n = 3.
(PPTX)
